# Advanced Signal Processing and Adaptive Learning Methods

**DOI:** 10.1155/2019/5428615

**Published:** 2019-11-03

**Authors:** Zoran Perić, Vlado Delić, Zoran Stamenković, David Pokrajac

**Affiliations:** ^1^University of Niš, Niš, Serbia; ^2^University of Novi Sad, Novi Sad, Serbia; ^3^IHP–Leibniz-Institut für Innovative Mikroelektronik, Frankfurt, Germany; ^4^Boeing, Everett, WA, USA

Research in the areas of signal processing and artificial intelligence (and developed methods and algorithms) has become increasingly important in the last two decades. In this special issue, we present new ideas and hybrid approaches based on techniques from the aforementioned scientific areas encouraging researchers from different fields to adopt them in accomplishing complex multidisciplinary tasks. This special issue includes a set of novel contributions, which covers a wide range of advanced signal processing techniques and adaptive learning methods for various engineering purposes.

In the paper entitled “RnRTD: Intelligent Approach Based on the Relationship-Driven Neural Network and Restricted Tensor Decomposition for Multiple Accusation Judgment in Legal Cases,” X. Guo et al. describe a new method for judging multiple crimes in legal cases. This is a multilabel classification, and it is based on the relationship-driven recurrent neural network (rdRNN) and restricted tensor decomposition (RTD). The authors have demonstrated that the proposed RTD layer and the relation-driven cyclic neural network have remarkable optimization effects on various deep neural network algorithms.

Next, a novel emotion identification method based on mutual information feature weight, which captures the correlation and redundancy of features, is presented in the paper entitled “Adaptive Learning Emotion Identification Method of Short Texts for Online Medical Knowledge Sharing Community” by D. Gan et al.

The paper entitled “Speech Technology Progress Based on New Machine Learning Paradigm” by V. Delić et al. provides an overview of speech technologies development as a typical signal processing area. The authors provide an analysis of the nature of speech signal and processing, corresponding machine learning algorithms, and applied computational intelligence in order to give an insight into several fields, covering speech production and auditory perception, cognitive aspects of speech communication and language understanding, both speech recognition and text-to-speech synthesis in more detail, and consequently the main directions in development of spoken dialogue systems. Additionally, the article discusses the concepts and recent advances in speech signal compression, coding, and transmission, including cognitive speech coding.

“Using Morphological Data in Language Modeling for Serbian Large Vocabulary Speech Recognition” is another paper in the area of speech signal processing, presented by E. Pakoci et al. In the paper, the authors have demonstrated that using additional morphological knowledge for language model training can solve a large part of problems for highly inflective languages such as Serbian.

A product module network design based on complex network theory is described in the paper entitled “Product Module Network Modeling and Evolution Analysis” by H. Qiao et al. In order to analyze the change in the product module network caused by module evolution, a BBV (Barrat–Barthelemy–Vespignani) model of the product module network is established to dynamically determine the brittle risk of the product module network.

“Steady-State Motion Visual Evoked Potential (SSMVEP) Enhancement Method Based on Time-Frequency Image Fusion” is the title of the paper by W. Yan et al., in which the authors propose an SSMVEP enhancement method based on T-F image fusion in order to explore whether T-F domain analysis can achieve better fusion effects than time-domain analysis. The authors have demonstrated that the key to the T-F image fusion algorithm is the fusion of the wavelet low-frequency components and that the online performance of the T-F image fusion method is better than that of the traditional spatial filtering methods, which indicates that the proposed method is feasible to fuse SSMVEP in the T-F domain.

The paper entitled “Deep Learning Network for Multiuser Detection in Satellite Mobile Communication System” by G. Q. Yang et al. proposes a multiuser detection (MUD) algorithm based on deep learning network for the satellite mobile communication system. The authors have demonstrated that through establishing a typical satellite communication system simulation platform, compared with the orthogonal signal tracking (OMP) and iterative sorting least squares (IORLS) algorithms, the proposed deep learning network algorithm has better performance in different conditions of SNR, CINR, and carrier frequency offset interference.

A novel methodology for increasing the predictions accuracy of different ANN-based systems is presented in the paper entitled “Concurrent, Performance-Based Methodology for Increasing the Accuracy and Certainty of Short-Term Neural Prediction Systems” by M. Milić et al. The authors have tested the method on GNI forecasting at national economy level, municipal traffic volume forecasting, and suburban daily electric load consumption forecasting.

The paper “KeratoDetect: Keratoconus Detection Algorithm Using Convolutional Neural Networks” by A. Lavric et al. proposes an algorithm facilitating the diagnostics of keratoconus disease. The algorithm analyzes the corneal topography of the eye using a convolutional neural network (CNN) that is able to extract and learn the features of a keratoconus eye. The authors have demonstrated that the algorithm can assist the ophthalmologists in rapid screening of patients, thus reducing diagnostic errors and facilitating treatment.

Finally, N.-D. Hoang proposes an automatic image processing approach for periodically evaluating the condition of wall structures in the paper entitled “Image Processing-Based Recognition of Wall Defects Using Machine Learning Approaches and Steerable Filters.” In order to extract useful features from digital images, steerable filters and projection integrals are employed. The author has demonstrated that the proposed method can achieve a good classification performance with a high classification accuracy, and as such, it represents a promising aid in periodic building surveys by maintenance agencies.

By providing a variety of applications, from traditional signal processing areas such as speech, image, and medical signal processing to the applications for analysis of legal matters and civil engineering, we have shown that whatever our primary research area is, machine learning and signal processing techniques can significantly improve the performance of existing methods and enable emersion of novel approaches. Application of such advanced techniques may also contribute to the progress in a number of other areas, which is an additional mission of this special issue.

